# Contact precautions for the control of endemic pathogens: Finding the middle path

**DOI:** 10.1017/ash.2023.145

**Published:** 2023-03-24

**Authors:** Gonzalo M. Bearman, Anthony D. Harris, Evelina Tacconelli

**Affiliations:** 1 Division of Infectious Diseases, Department of Medicine, Virginia Commonwealth University, Richmond, Virginia, United States; 2 Department of Epidemiology and Public Health University of Maryland School of Medicine, Baltimore, Maryland, United States; 3 Division of Infectious Disease, Department of Diagnostics and Public Health, University of Verona, Verona, Italy, for the ESCMID European Committee on Infection Control (EUCIC), Basel, Switzerland

“As the man said, for every complex problem there’s a simple solution, and it’s wrong.”

— Umberto Eco, *Foucault’s Pendulum*

The control of endemic pathogens, such as methicillin-resistant *Staphylococcus aureus* (MRSA), vancomycin-resistant enterococci (VRE) and extended-spectrum β-lactamase–producing Enterobacterales (ESBL-E), is evolving and controversial. The incremental benefits of contact precautions for the control of endemic pathogens beyond standard precautions are not clearly defined. Unlike the physical sciences, the science of infection prevention is inexact and not easily assessed in controlled environments because cluster randomized trials are expensive and logistically challenging, likely requiring >100 hospitals to reach statistical significance.^1^

The prevention of infection in healthcare settings is nuanced and is driven by many factors: resistance patterns in hospitals, long-term care facilities and community, healthcare worker compliance with basic and advanced infection control practices, antibiotic consumption, case-mix index of patients, severity of patients’ comorbidities, colonization pressure of wards, the inanimate environment, microbiology laboratory capability, human behavior, and the multiple complex healthcare-worker patient interactions at the bedside. Accurate assessment of the benefits of contact precautions is also strictly linked with implementation and compliance with other infection control interventions such as universal or targeted screening, pre-emptive isolation, and placing patients in cohorts.

We explored different perspectives for the use of contact precautions in settings endemic for antibiotic-resistant bacteria (ie, colonization and/or infection), and we sought a middle-path solution to a complex problem.

## Universal gloves and gowns

A large, cluster-randomized trial provides much of the highest-level evidence relative to the benefits of contact precautions for certain antibiotic-resistant bacteria.^2^ This trial was conducted in 20 adult medical and surgical intensive care unit (ICUs). We compared the effect of universal glove and gown use for all patient contact and when entering any patient room with standard practice (ie, the use of gowns and gloves only for patients known to be infected or colonized with antimicrobial-resistant bacteria) on the rate of acquisition of antimicrobial-resistant gram-positive organisms. Overall, 26,180 patients were included in the study. Although the investigators found no difference between the 2 arms in the primary outcome of acquisition of either MRSA or VRE, there was a significantly greater relative reduction in the prespecified secondary outcome of MRSA acquisition in intervention units compared to control units (40.2% vs 15%; *P* = .046). The intervention led to a decrease in MRSA acquisition and no effect on VRE acquisition. A weakness of the study is that it was underpowered to study healthcare-associated infection rates. In subsequent studies of stored perirectal specimens that were part of the original study, the intervention showed no effect on decreasing *Clostridium difficile* acquisition but did show a trend toward decreasing antibiotic-resistant Gram-negative bacteria acquisition especially *Acinetobacter* and Pseudomonas species.^3,4^

For multidrug-resistant gram-negative bacteria, there is general agreement among international evidence-based guidelines on a strong recommendation in implementing contact precautions, although based on a low level of evidence, for reducing intrahospital transmission of *Klebsiella pnemoniae*, *Acinetobacter baumannii*, and *Pseudomonas aeruginosa* resistant to carbapenems.^5–7^ However, the management of hospitalized patients colonized or infected with ESBL-E is under debate. No clear evidence is available for benefit of contact precautions beyond standard precautions for the control of all ESBL-E species. For some strains, such as *Escherichia coli*, there is evidence that in-hospital transmission might not be the primary driver of the hospital spread. For non–*E. coli* spp, environmental contamination is more frequently reported than with *E. coli* and might serve as a secondary reservoir for cross transmission.^8^ In a cluster-randomized crossover trial in 20 non-ICU wards in 4 European university hospitals, patients colonized with ESBL-E were randomized to contact precautions alone versus standard precautions for 12 months. More than 11,000 patients were screened at least twice. The incidence density of ward-acquired ESBL-E was not statistically different between the 2 periods: 6.0 events per 1,000 patient days at risk during periods of contact precautions and 6.1 during standard precautions. Multivariable analysis adjusted for length of stay, percentage of patients screened, and prevalence in first screening cultures yielded an incidence rate ratio of 0.99 (95% CI, 0.80–1.22; *P* = .9177) for care under contact precautions compared with standard precautions).^9^ The study had several limitations. There were delays between patient surveillance sampling and result notification from the microbiology to the ward in nearly two-thirds of patients with ESBL-E. Also, 50% of single-occupancy rooms had ESBL-E. These factors could have acted as confounders in the assessment of hospital cross transmission. Based on the currently available evidence, the decision to implement contact precautions in patients colonized or infected with ESBL-E should be locally determined based on species involved and setting (eg, risk for potential transmission in wards with fragile populations).

## The de-escalation of contact precautions in endemic settings

In 2015, Morgan et al^10^ published a survey of the Society for Healthcare Epidemiology of America research Network (SHEA RN) reporting that 60% of epidemiologist interviewed were interested in an alternative option to contact precautions for the control of endemic pathogens. In addition, at the time of publication, >30 US hospitals were not employing contact precautions for the control of endemic pathogens such as MRSA or VRE.^11^ Reports of de-escalation of contact precautions in endemic settings exist in the literature. Using a before-and-after analysis, a single-center study reported a 45% reduction in contact precaution burden following de-escalation, with no change in the already decreasing rates of MRSA, VRE and all pathogen National Healthcare Safety Network (NHSN) device-associated infections.^12^ This was also associated with a $700,000 annual cost saving in personal protective equipment. An additional publication replicated these findings in 2016, using a similar study design, with similar cost reduction and no negative impact on all decreasing NHSN-defined healthcare-associated infections.^13^ Similar outcomes with the de-escalation of contact precautions for endemic MRSA or VRE colonized and infected patients is reported in the pediatric literature.^14^

In a single-center, interrupted time-series analysis over a 7-year period, the de-escalation of contact precautions for endemic MRSA and/or VRE colonized and infected patients did not negatively impact already decreasing trends of all healthcare associated infections.^15^ In a multicenter interrupted time-series analysis, across 3 academic medical centers, over a 15-year period, the de-escalation to contact precautions for endemic MRSA and VRE infections resulted in no negative impact on the rates of NHSN-defined device-associated infections and mediastinal surgical-site infections.^16^ More recently, in an interrupted times-series analysis across 15 acute-care hospitals, the discontinuation of contact precautions resulted in no healthcare-associated infection rate increases.^17^ In the recent editorial published in the *Journal of Infection Prevention*, Wilson and Prieto^18^ concluded that the incremental benefit of contact precautions beyond standard precautions for the control of endemic pathogens is lacking and should be subjected to thorough and robust review to better define evidence-based recommendations.

Studies reporting the impact of contact precautions de-escalation have limitations. The major limitation is that these studies were underpowered to the outcome of infection rates. In addition, many of these studies were conducted in settings of additional, bundled interventions adopted during the period that contact precautions stopped. The proportionate impact of the bundled prevention components on infection outcomes is unclear. In addition, most are single-center design with either a before-and-after analysis or are quasi-experimental with an interrupted time-series analysis model. Studies do not report on colonization pressure and do not report postdischarge surveillance for MRSA or VRE-colonized and -infected individuals. In addition, current reports are from resource-rich environments, with a preponderance of single-occupancy rooms. Compliance with process-of-care measures varied across sites where contact precautions were de-escalated. These data may not be applicable to all patient care environments, particularly where there is a predominance of multiple-occupancy rooms.

## Concerns about contact precautions and adverse events

Numerous studies have attempted to address whether contact precautions lead to an increase in adverse events.^19–22^ Some observational studies have shown an increase in adverse events including increased depression, anxiety, falls, and electrolyte disorders as well as decreased patient satisfaction.^19,20^ However, most of these studies did not control for comorbidity of patients and severity of illness of patients and thus suffer from confounding by indication. The only randomized trial to assess whether contact precautions led to more adverse events showed a lower frequency of healthcare workers visits per hour (4.28 vs 5.24; *P* = .02) in ICUs using gowns and gloves for contact with all patients compared with control ICUs using gowns and gloves only for patients known to be colonized or infected with antimicrobial-resistant organisms and as otherwise required for CDC-defined contact precautions. The incidence of adverse events, however, was not significantly different between the 2 groups. Rates of preventable, nonpreventable, severe, and nonsevere ICU adverse events were actually all nonsignificantly lower in the contact-precaution intervention group.^2^ Thus, the highest-quality study did not support the hypothesis that contact precautions lead to an increase in adverse events.

## The middle path: A precision-based application of contact precautions in endemic settings

**Fig. 1.**
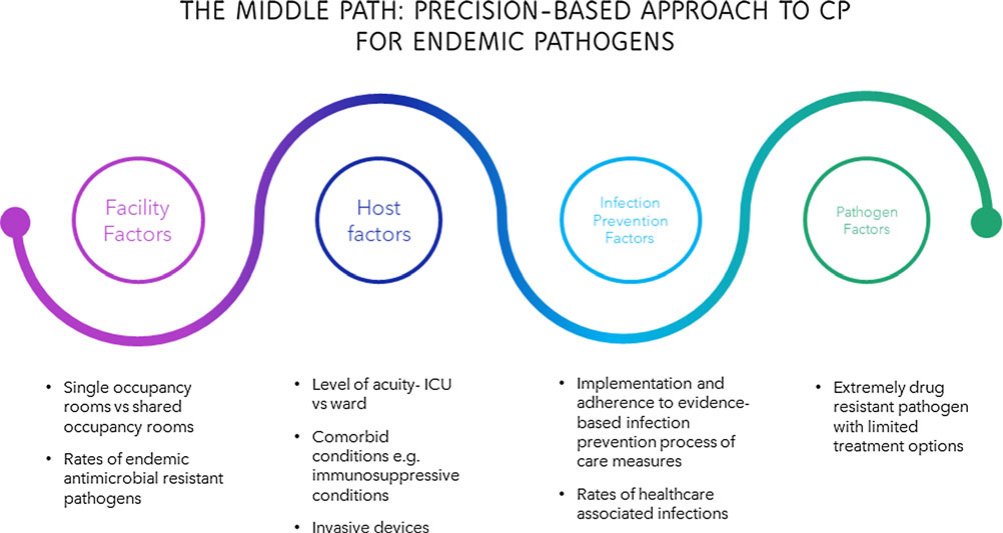
The middle path: precision-based approach to contact precautions (CP) for endemic pathogens.

A nuanced approach for the application of contact precautions in endemic settings with considerations for a more patient-centered, precision-based application of patient isolation is required (Fig. 1). This approach also includes a reassessment of implementation of other infection control measures, such as presurgical screening for locally epidemiologically important pathogens and related pre-emptive isolation, which are strictly linked with contact precautions. The goal is to judiciously apply contact precautions for the highest-risk populations (ie, patient individual risk and species potential for transmission) with the greatest efficiency; to minimize overuse of gloves and gowns; and to limit healthcare worker annoyance to the donning and doffing of personal protective equipment (and therefore risk of low compliance). The middle-path approach entails avoiding blanket recommendations of contact precautions driven solely by the presence of a specific antimicrobial resistance pattern and/or by the bacteria local endemicity. Instead, the middle-path approach recommends contact precautions by applying a patient-centered, multimodal assessment of the following elements: efficacy of contact precautions by patient comorbidities, healthcare procedures needed for clinical and surgical management of patients, type of interactions with healthcare workers, and local epidemiological data.

Recognizing the shortcomings in the one-size-fits-all approach for contact precautions is the immediate first step. For those in the “no contact precaution camp,” the use of gloves and gowns only in ICUs or for high-acuity patients (eg, left-ventricular-assist–device patients and extracorporeal membrane oxygenation patients and patients with multiple lines or those recently transplanted or under chemotherapy) may be of benefit. On the other hand, for those in the “contact precautions for everyone camp,” contact precautions are likely overused in low-risk settings such as ambulatory clinics or lower-risk locations like behavioral health and single-occupancy rooms and wards without a preponderance of patients who are immunocompromised or who have multiple invasive devices. Hospitals that still require gowns and gloves for visitors are likely overusing contact precautions.

The difficulty in implementing a more nuanced and patient-centered contact precautions policy should not be a barrier. The overarching goal is a shift toward newer infection control policies that prevents transmission and subsequent infection to those most at risk, decreasing annoyance with extensive usage of gloves and gowns, improving adherence to standard and contact precautions, and diminishing hospital costs. Thus, the focus of infection control teams in terms of local implementation of contact precautions should be a more “precision medicine–based” approach, with use of gloves and gowns reserved to those patients for whom the benefits are clear. This approach then requires a preliminary assessment of setting (ie, patient, bacterial ecology, and environment), dedicated education initiatives for healthcare workers, and ongoing assessment of compliance with standard precautions, gown and glove use, and surveillance of infections caused by antibiotic-resistant bacteria.

Further assessment of patient-centered outcomes, including safety events, is also required. This element is critical to understanding not only patient-centered infection prevention outcomes but also the impact of contact precautions on other safety events such as patient falls, perceptions of isolation, and satisfaction with care. Last, the ongoing application of contact precautions requires a cost analysis as well as further exploration of the environmental impact of excess gown and glove use.

A one-size-fits-all application of contact precautions for the control of endemic pathogens is unsatisfactory for all patient populations. There is an urgent need to raise decision making on implementation of contact precautions to a higher level that employs a more precision-based and patient-centered approach, one coupled with the relentless pursuit of standard precautions and sustained hand-hygiene practice across health systems.
